# Editorial: Multi-omic Data Integration in Oncology

**DOI:** 10.3389/fonc.2020.01768

**Published:** 2020-09-15

**Authors:** Francesca Finotello, Enrica Calura, Davide Risso, Sampsa Hautaniemi, Chiara Romualdi

**Affiliations:** ^1^Biocenter, Institute of Bioinformatics, Medical University of Innsbruck, Innsbruck, Austria; ^2^Department of Biology, University of Padua, Padua, Italy; ^3^Department of Statistical Sciences, University of Padua, Padua, Italy; ^4^Research Program in Systems Oncology, Faculty of Medicine, University of Helsinki, Helsinki, Finland

**Keywords:** multi-omic, single-cell, transcriptomics, pathways, cancer, data integration

In the next few years, we are going to witness changes in the treatment of cancer patients due to molecular and personalized medicine. Indeed, many hospitals are already starting routine genome-wide screening to complement and inform diagnosis and treatment choices. However, the majority of molecular aberrations identified in cancers have synergic interactions in many aspects of cell signaling beyond the genome. The complexity of cancers cross cell boundaries especially studying the tumor microenvironment as a heterogeneous and dynamic network of interacting cells (1), one of the new hot topics for anticancer treatment development. In this scenario, multi-omic technologies and single-cell data can shed light on these interactions by generating high-throughput datasets portraying the genomes, transcriptomes, proteomes, metabolomes, and epigenomes of tumors.

Large-scale cancer genomic projects, such as The Cancer Genome Atlas (TCGA) (2), have generated petabytes of multi-omic data portraying this heterogeneity. Importantly, these data have been made available to the scientific community, shifting the main challenge from data collection to data analysis and integration, and allowing for development of novel data analysis methods. However, while computational and statistical analyses of single-omics datasets are well-established—excluding the still challenging single-cell data analyses—the integration of multi-omic data is still far from being standardized. As the number of datasets grows and the biological knowledge increases, existing methods should be extended or generalized, and new computational tools need to be proposed to cope with the complexity and multi-level structure of the available information. In this special issue, de Anda-Jáuregui and Hernández-Lemus presented a comprehensive review of the state of the art of multi-omic data analysis in oncology, encompassing a wide range of tasks, such as data acquisition and processing, data management, identification of therapeutic targets, as well as patient classification, diagnosis, and prognosis.

One of the major challenges in the analysis of multi-omic data is how to integrate the different data modalities. Nicora et al. reviewed a selection of recent tools for the computational integration of multi-omic data sets based on: deep learning, network integration, data clustering or factorization, and feature extraction or transformation. This emerging field has already contributed a rich catalog of freely available tools: the most widely used approaches are network-based methods, but deep learning strategies are becoming increasingly popular. In this context, Chierici et al. proposed a computational framework for high-throughput data integration (called Integrative Network Fusion, INF), which leverages network structures and machine learning models to extract multi-omic predictive biomarkers for cancer subtype identification. By integrating gene expression, protein expression, and copy-number data across three TCGA cancer types, INF showed a higher predictive performance with respect to simple juxtaposition of single-omics analyses and enabled the extraction of more biologically meaningful biomarkers. INF was designed to integrate an arbitrary number of omic layers, allowing to extend the framework to other types of data, such as histopathological and radiological images.

The main goal of most integrative methods is the identification of multi-omic signatures that can be diagnostic (healthy vs. disease), prognostic (good vs. poor patient outcome), or predictive (good vs. poor response to therapeutic interventions). The selection of the optimal signature size, that is the number of molecular features needed to stratify patients, is not trivial. In general, the smaller the signature size, the easier its clinical applicability, but the lower its accuracy, due to patients heterogeneity. In this perspective meta-analysis studies that exploit data from previously published studies can increase the signature robustness and reliability. Liu et al. combined extensive text mining and transcriptomic data to identify and validate a small prognostic signature in liver cancer. By selecting more than thousand genes known to be involved in liver cancer initiation and progression, they identified a triplet of genes associated with survival. Using three independent cohorts and specific experimental assays to confirm transcript and protein expression levels, they found that low expression of *F2, GOT2*, and *TRPV1* is associated with poor prognosis in liver cancer. In a parallel study, Li et al. identified a small diagnostic signature composed of long non-coding RNAs (RP11-33A14.1, RP11-423H2.3, and LAMTOR5-AS1) that, combined with clinical and previously-published molecular biomarkers, is able to predict prostate cancer from fine needle aspiration biopsies with high sensitivity and specificity. Looking for potential molecular functions of the signature elements, the authors suggested and validated a sponge mechanism, that sees miR-7, miR-24-3p, and miR-30 as the three main miRNAs sequestered by the long non-coding RNAs, which in turn interact with the RNA binding protein FUS.

While the identification of precise molecular signatures is fundamental for clinical practice, the understanding of the actual mechanisms driving these alterations in specific cancers or cancer subtypes is crucial to design new pharmacological treatments. Ochoa et al. investigated the regulatory elements that drive the various expression behaviors of the PAM50 signature (3) in different breast cancer subtypes. The authors integrated coding and non-coding gene expression, methylation levels, and information on transcription factors (TF)-target interaction data via a generalized elastic-net model. Using breast tumors and normal adjacent tissues from the TCGA, they identified both subtype-specific regulators and regulators acting across subtypes, such as miR-21 and miR-10b. With a similar aim, Tait et al. combine transcriptomic data to study the expression patterns of non-coding elements (miRNAs and long non-coding RNAs, ncRNA) underlying dysfunctional adipocyte phenotype in obesity and colorectal cancer. The authors inferred lncRNA-miRNA-mRNA modules, highlighting several ncRNA modulations and dysregulated pathways that are common to both obesity and colorectal cancer. Chen et al., using whole exome and transcriptome sequencing, studied the genomic and transcriptomic landscape of cholangiocarcinoma. The authors investigated subnetworks that were greatly influenced by tumor clonal or subclonal mutations impacting gene expression.

Immunotherapy with checkpoint blockers has drastically advanced treatment of different types of cancer over the past years, improving overall patient survival compared to standard therapy. However, response to treatment remains hard to predict due to the large intra- and inter-patient heterogeneity. Lapuente-Santana and Eduarti reviewed the benefit of multi-omic approaches for biomarker discovery in the immuno-oncology field. They present multi-omic approaches that could help understand how different immune cell types can influence the efficacy of immunotherapy with checkpoint blockers and how the cells interact in the tumor microenvironment, shaping the immune response, and resistance to immunotherapy. The authors suggest that a combination of dynamic mathematical models and longitudinal data could further improve our understanding of the tumor microenvironment role in the response to immunotherapy and provide the rationale for alternative personalized treatments.

Another field that recently had a boost from multi-omic integration strategies is pharmacogenomics. The term pharmacogenomics is generally used to define the variability of drug response due to the patients' genomic landscape. In this context, cancer cell lines have been the most widely used models to explore the molecular basis of drug sensitivity. Starting from the first NCI-60 project (4), several other studies investigating the link between the genomic makeup and drug response in cancer cell lines have been carried out (5–7). Caroli et al. reviewed the databases and computational tools that have been developed to integrate cancer cell lines genomic profiles and sensitivity to small molecule perturbations obtained from different screenings.

Multimodal omics can be integrated *in silico* to respond to complex biological questions that require a systems biology approach. One of such examples is the prediction of tumor neoantigens, namely mutated peptides that are bound to the major histocompatibility complex molecules of cancer cells and can elicit anticancer immune responses. Schrörs et al. derived an integrated map of the genome, transcriptome, and neoantigen landscape of one of the most widely used breast cancer models: the 4T1 murine mammary cancer cell line. They found that 4T1 cells share molecular features with triple-negative breast cancer and, thus, represent a promising model for preclinical studies. Moreover, the authors confirmed experimentally the antigenic potential of 23 mutated peptides selected from the pool of neoantigens predicted *in silico* using IFNγ-ELISpot assays.

Despite their recognized value to advancing and informing immuno-oncology and precision medicine, standard “bulk” technologies are intrinsically limited by the sequencing of heterogeneous cell mixtures, which renders a blended average portrayal of the tumor microenvironment. Rapidly-emerging single-cell technologies allow to disentangle the phenotypes of individual cells, providing unprecedented insights into the cellular and spatial diversity of the tumor microenvironment. However, the sparsity, noise, and high-dimensionality of single-cell data pose unique challenges to data analysis. Hsu and Culhane provide a guide to dimensionality reduction techniques that are vital to extract the major sources of variations from single-cell RNA-sequencing data prior to performing downstream data integration, clustering and analysis. The authors focused on principal component analysis (PCA), a matrix factorization method that can easily scale to large datasets when used with sparse-matrix representations; they described its relationship with singular value decomposition, the differences between using correlation or covariance matrices, the impact of data scaling, log-transformation, and standardization, and how to recognize artifacts in PCA plots. Moreover, they described how canonical correlation analysis (CCA), another popular matrix factorization approach, can be used to integrate single-cell data from different platforms or studies.

Despite their promise, single-cell technologies, such as flow cytometry, mass cytometry, or single-cell RNA sequencing, are still limited by the lack of information on spatial context and multicellular interactions. de Vries et al. show how multimodal and spatially-resolved single-cell data can advance our understanding of the inter-cellular organization and communication in the tumor microenvironment. They present recent developments in spatial, tissue-based techniques, such as multiparameter fluorescence, imaging mass cytometry, and *in situ* transcriptomics, as well as, multidimensional single-cell technologies and studies that integrate multiple single-cell modalities to disentangle complex cell interactions in the tumor microenvironment. These approaches hold the promise to uncover the sources of intra-tumor heterogeneity that hamper cancer treatment but require the development of dedicated bioinformatic tools for the data analysis and interpretation and tight collaboration between oncologists, immunologists, pathologists, and bioinformaticians for the extraction of mechanist rationales and actionable targets.

Overall, our collection of original research articles and reviews covers a wide range of multi-omic applications in oncology. The scenario that emerges is that transcriptomics, methylomics, and genomics are the three most frequently analyzed and integrated data, both in bulk and single-cell studies. To fully understand the complex interactions of the molecular processes underlying cellular mechanisms a fine temporal and spatial resolution is required. Spatial transcriptomics (8), a set of techniques that allow the (sub-) cellular characterization of gene expression, has the potential to unveil the complex interplay between cell types but gives rise to new computational and statistical challenges, also in terms of data integration. In addition, important information can be exploited by integrating omics data and biomedical images (9), a field that is experiencing new advances in terms of sensitivity and resolution. Multi-modal integrative analysis will soon become the standard to study complex systems, and we look forward to exciting new computational developments to tackle data heterogeneity, computational efficiency and results interpretation, and can ultimately push the oncology field forward.

## Author Contributions

All authors listed have made a substantial, direct and intellectual contribution to the work, and approved it for publication.

## Conflict of Interest

The authors declare that the research was conducted in the absence of any commercial or financial relationships that could be construed as a potential conflict of interest.
